# Recent Travel and Tuberculosis in Migrants: Data From a Low-Incidence Country

**DOI:** 10.1093/cid/ciad672

**Published:** 2023-11-06

**Authors:** Alvaro Schwalb, Kumvana Kayumba, Rein M G J Houben, Graham H Bothamley

**Affiliations:** TB Modelling Group, TB Centre, London School of Hygiene and Tropical Medicine, London, United Kingdom; Department of Infectious Disease Epidemiology, London School of Hygiene and Tropical Medicine, London, United Kingdom; Instituto de Medicina Tropical Alexander von Humboldt, Universidad Peruana Cayetano Heredia, Lima, Peru; Department of Infectious Disease Epidemiology, London School of Hygiene and Tropical Medicine, London, United Kingdom; TB Modelling Group, TB Centre, London School of Hygiene and Tropical Medicine, London, United Kingdom; Department of Infectious Disease Epidemiology, London School of Hygiene and Tropical Medicine, London, United Kingdom; Faculty of Infectious and Tropical Diseases, London School of Hygiene and Tropical Medicine, London, United Kingdom; Department of Respiratory Medicine, Homerton University Hospital, London, United Kingdom

**Keywords:** tuberculosis, migrant health, epidemiology, travel, infection

## Abstract

Tuberculosis (TB) incidence rates among migrants are higher than those in low-incidence countries. We evaluated smear-positive, pulmonary TB notifications of foreign-born individuals, comparing time since arrival and time since last return travel to the country of origin. TB incidence suggests a time course consistent with recent infection during travel.

## BACKGROUND

Migrants from countries with a high incidence of tuberculosis (TB) develop disease at similar rates to their country of origin rather than the incidence among people born in the United Kingdom [1]. In 2020, the TB incidence in foreign-born individuals was 36.3 per 100 000 inhabitants, a rate substantially higher in the UK-born population [2]. Foreign-born individuals account for over 70% of all TB notifications, which is above the median for high-income countries [2, 3].

The high TB burden among migrants can be explained in several ways: active disease present on arrival, local transmission (via increased contact with individuals who speak the same language, have similar cultures, etc.), or disease progression due to the high prevalence (≈37%) of *Mycobacterium tuberculosis* (*Mtb*) infection [3]. However, the acquisition of *Mtb* infection following exposure during travel likely plays a role in TB epidemiology in the migrant population [3]. TB notification data show that a quarter of foreign-born individuals had travelled abroad for more than a month in the two years prior to TB diagnosis, a higher proportion than those born in the United Kingdom [2]. As longitudinal studies support an incubation period for disease progression of up to two years, it is worth evaluating if recent exposure via travel matches this proposed timeline [4]. We sought to compare TB notifications of foreign-born individuals using time since arrival to the United Kingdom and time since most recent travel.

## METHODS

We assessed TB notifications in foreign-born adults over a 20-year period in the London Borough of Hackney, United Kingdom. The population of Hackney is ethnically diverse, with over 73 languages spoken, and 39% of its residents being foreign-born [5]. In 2018, the incidence rate of TB was 19.1 per 100 000 inhabitants, a substantial decrease from 62.7 reported in 2000 [2]. Notification data were routinely collected within the TB service at the Department of Respiratory Medicine at Homerton Healthcare NHS Foundation Trust from 1996 through 2018 and audited to improve early diagnosis and access to health care. The hospital serves Hackney's population and offers both hospital and community care. Individuals were made known to the TB service through general practice, the emergency and radiology department, internal referrals, and various screening clinics, including migrant, contact tracing, occupational health, homeless shelters, and human immunodeficiency virus (HIV)-positive cases.

Only individuals with smear-positive, culture-positive pulmonary TB (S + PTB) were considered for this analysis as representing the greatest potential for onward transmission of *Mtb*. This restriction prevented overdiagnosis of TB in a population considered at risk of disease without microbiological confirmation, variation related to the time of evolution in the spectrum of TB disease and allows comparison with similar studies. We excluded individuals with prior active TB or preventive therapy. We recorded information on most recent travel, which included either a high TB burden country (usually country of origin of individual or spouse) or to a high-risk setting, and time of entry to the United Kingdom for the index case. A high TB burden country was defined as having an incidence rate exceeding 150 cases per 100 000 inhabitants in the year of diagnosis. When unable to provide an exact date, a range was noted, and the earliest time was analyzed. We used the time of the first contact with the healthcare system prompted by TB symptoms (cough, sputum, hemoptysis) or following abnormal imaging (often due to fever, night sweats or positive tuberculin skin test, and/or interferon-gamma release assay screening) as a proxy for diagnosis time to avoid variations caused by the diagnostic process and notification delay to public health authorities. We constructed TB diagnosis time curves to compare recent return to the United Kingdom after travel and time since first arrival in the United Kingdom.

The study was deemed an audit by the National Institute for Health and Care Research and by the Research and Innovation Office at Homerton Healthcare NHS Foundation Trust. Ethical approval was granted by the LSHTM Ethics Committee (ID:25968) to analyse the anonymised data. Data analysis utilized R v.4.2.2 for statistical computing and graphics.

## RESULTS

Data were available for a total of 369 migrants with S + PTB. We excluded 25 (7%) individuals with no information on time since migration or travel. Additionally, 24 (7%) individuals were part of an isoniazid-resistant *Mtb* strain outbreak, indicating local transmission, and were also excluded. Of the remaining 320 individuals, most were male (n = 216, 68%) and over half (n = 191, 60%) were aged 16 to 35. The median duration of travel was 31 days (interquartile range [IQR]: 28–55). Active case-finding identified 26 (8%) cases. Regarding risk factors for TB disease progression, diabetes, high alcohol consumption, and homelessness were reported in 34 (11%), 31 (10%), and 26 (8%) individuals, respectively. HIV-positive serostatus was confirmed in 26 (8%) individuals; it was unknown for 113 (35%) at the time of TB diagnosis.

**Figure 1.**
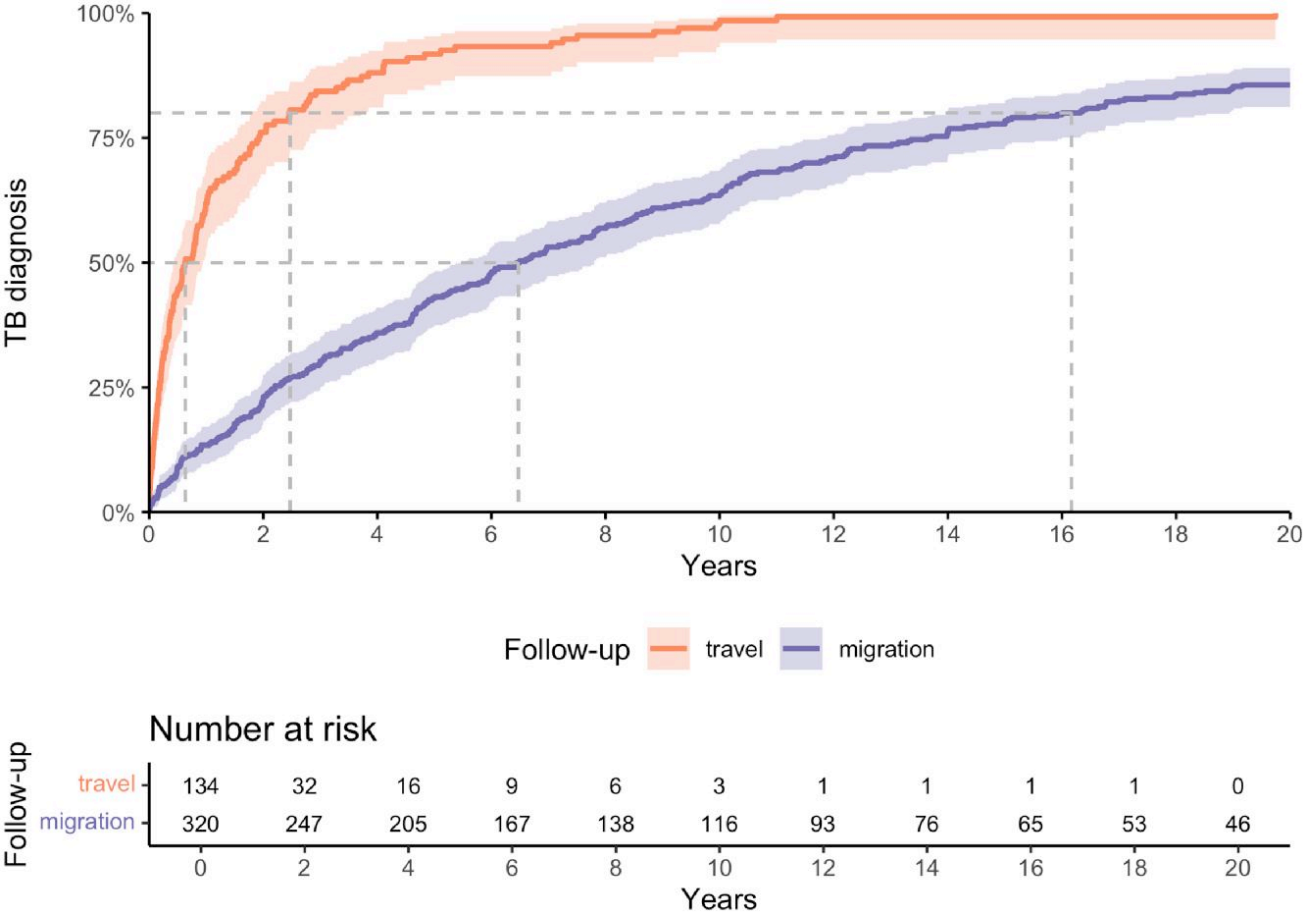
Tuberculosis diagnosis from the time since migration and recent travel in a migrant population in the London Borough of Hackney. Migration from and travel to a high-burden TB setting. Abbreviation: TB, tuberculosis.

A total of 134 (42%) individuals reported travelling since first migrating to the United Kingdom, with 89 (66%) travelling to high TB burden countries (Supplementary Table 1). Following the curve for the individuals with recent travel (Figure 1), 50% of TB diagnoses occurred within the first year (0.63 years; 95% confidence interval [CI]: 0.44–0.94) and 80% within three years (2.47 years; 95% CI: 1.80–3.72) after their return. Conversely, when considering the time from migration to the United Kingdom as the starting point, 50% and 80% of TB cases were diagnosed after five years (6.47 years; 95% CI: 5.50–7.80; and 14 years (16.16 years; 95% CI: 14.00–18.93), respectively. In a sensitivity analysis, we restricted the sample to only those who reported travel, as that would show the clearest reflection of progression after exposure (Supplementary Figure 1). A constant rate is observed focusing on time since migration whereas the time since recent travel strongly suggests that most disease occurred by the second year. In another sensitivity analysis, we excluded individuals that were identified as part of active screening procedures, as this would result in a decrease in time to diagnosis. No significant changes were observed in the time to 50% of TB diagnoses, which were 0.76 years (95% CI: 0.48–0.99) and 6.63 (95% CI: 5.60–7.84) for time since recent travel and migration, respectively (Supplementary Figure 2). Finally, two sensitivity analyses examined travel exclusively to high TB burden countries (Supplementary Figure 3) and excluded individuals who travelled to low TB burden countries (Supplementary Figure 4).

## DISCUSSION

Although TB disease among migrants has been traditionally thought to result from late progression following infection before arrival, our findings suggest that recent exposure plays an important role in the development of S + PTB. Another study also indicated that TB primarily occurs in recent (<2 years) entrants to the United Kingdom, likely driven by exposure shortly before migration [6]. Other studies have shown that 15%–50% of TB incidence among migrants could be due to travel back to the country of origin to visit friends and relatives [7]. Previously, a study in the Netherlands alluded to the increased travel-associated odds, particularly when the duration of travel exceeded three months [8]. This is evident when using the time since recent travel as the starting point for assessing disease progression, rather than the original time of migration into a low-incidence country. If the high TB rate among migrants is indeed driven by recent exposure, incidence curves using recent travel as follow-up start should better reflect estimated rates of disease onset post-exposure, as demonstrated here [9].

Our findings highlight recent travel to a high-burden country as a relevant question and risk factor. Although we were unable to calculate the proportion of TB incidence attributable to travel because our sample only comprised individuals with TB, the results show that, among those with a diagnosis after two years since migration, half had reported travel to their country of origin.

There are some limitations to address. This study is focused on the progression to S + PTB, which often is diagnosed more easily compared to smear-negative pulmonary or extra-pulmonary TB, which may involve lengthier diagnostic processes. To account for potential diagnostic delays, we opted to use the time since the first contact with the healthcare system. However, this approach restricts our insights to only S + PTB and does not capture progression to subclinical disease. Furthermore, travel was recorded dichotomously, whereas enriched travel histories that include duration and purpose (e.g., visiting relatives in a high-risk setting such as hospitals), and, ultimately, the contact rate would enable a more refined analysis, and exploration of a dose-response relationship [10]. On the other hand, although we have proposed that local transmission is less common among migrants, transmission could have occurred from relatives visiting the United Kingdom, which are not later identified as part of a cluster in outbreak studies once they return home. Overall, our findings warrant the inclusion of recent return travel to a high incidence country as a factor in TB risk predictor models [11].

Our study adds to the growing literature on TB burden among migrant populations in low-burden settings [3, 10]. Although we argue that travel to a high TB burden or high-risk region is a risk factor to be considered during clinical appraisal, research should also focus on the effectiveness of migrant-sensitive and friendly services to mitigate the risk of exposure during travel [10]. Concurrent inquiries about travel and TB at healthcare visits may improve the health of the migrant population.

## Supplementary Data

Supplementary materials are available at *Clinical Infectious Diseases* online. Consisting of data provided by the authors to benefit the reader, the posted materials are not copyedited and are the sole responsibility of the authors, so questions or comments should be addressed to the corresponding author.

## Supplementary Material

ciad672_Supplementary_Data

## Notes

***Author Contributions*.** Conceptualization: G. H. B. and R. M. G. J. H.; Data collection: G. H. B. Data curation: A. S., K. K., and G. H. B. Formal analysis: A. S. and K. K. Writing—original draft: A. S. Writing—review and editing: K. K., R. M. G. J. H., and G, H. B. Supervision: R. M, G. J. H. and G. H. B.

***Acknowledgments*.** The authors thank the Homerton University Hospital TB team who supported data collection and the original audit.

***Data availability*.** Data available at LSHTM Data Compass (doi: 10.17037/DATA.00003534) and analysis code at GitHub (https://github.com/aschwalbc/TBMigrants). Data have been restricted to fields needed to run main and sensitivity analyses.

***Financial support*.** This work was supported by the European Research Council (grant number 757699 to A. S. and R. M. G. J. H.) and the Fogarty International Center of the National Institutes of Health (grant number D43TW009763 [Program for Advanced Research Capacities for AIDS in Peru] to A. S.).
