# Supplementary material for: Recent Travel and Tuberculosis in Migrants: Data From a Low-Incidence Country
Source: Clin Infect Dis. 2023 Nov 6;78(3):742–5. doi: 10.1093/cid/ciad672 (PMC10954334; doi:10.1093/cid/ciad672)
Supplement: ciad672_Supplementary_Data [file ciad672_supplementary_data.docx]

**SUPPLEMENTARY MATERIAL:**

**Recent travel and tuberculosis in migrants: data from a low incidence country**

Alvaro Schwalb^1,2,3^, Kumvana Kayumba^2^, Rein M.G.J. Houben^1,2^, Graham H. Bothamley^4,5^

*^1^TB Modelling Group, TB Centre, London School of Hygiene and Tropical Medicine, London, United Kingdom; ^2^Department of Infectious Disease Epidemiology, London School of Hygiene and Tropical Medicine, London, United Kingdom; ^3^Instituto de Medicina Tropical Alexander von Humboldt, Universidad Peruana Cayetano Heredia, Lima, Peru; ^4^Faculty of Infectious and Tropical Diseases, London School of Hygiene and Tropical Medicine, London, United Kingdom; ^5^Department of Respiratory Medicine, Homerton University Hospital, London, United Kingdom*

**Correspondence:** Graham H. Bothamley, Department of Respiratory Medicine, Homerton University Hospital, London, E9 6SR, United Kingdom ([g.bothamley@nhs.net](mailto:g.bothamley@nhs.net))

**Table S1 – Countries and frequencies of travel.**

| *ISO3* | *Country of birth* | *Frequency* | *TB burden* | *Travel other than COB* |
| --- | --- | --- | --- | --- |
| AFG | Afghanistan | 1 | High |  |
| DZA | Algeria | 3 |  |  |
| AGO | Angola | 3 | High |  |
| BGD | Bangladesh | 10 | High |  |
| BRA | Brazil | 1 |  |  |
| CMR | Cameroon | 3 | High |  |
| CAN | Canada | 1 | Low | Travelled widely throughout SE Asia |
| CYP | Cyprus | 1 | Low | Incidence same as Türkiye |
| COD | D R Congo | 1 | High |  |
| ERI | Eritrea | 1 | High |  |
| ETH | Ethiopia | 3 | High |  |
| GMB | Gambia | 4 | High |  |
| GHA | Ghana | 3 | High |  |
| GNB | Guinea-Bissau | 1 | High |  |
| IND | India | 10 | High |  |
| ITA | Italy | 1 | Low | Exposure to high-risk community |
| JAM | Jamaica | 5 | Low | Other high-risk characteristics |
| KEN | Kenya | 1 | High |  |
| XKX | Kosovo | 1 |  |  |
| LBR | Liberia | 1 | High |  |
| MWI | Malawi | 1 | High |  |
| MUS | Mauritius | 1 |  |  |
| MSR | Montserrat | 1 | Low |  |
| MAR | Morocco | 1 |  |  |
| MMR | Myanmar | 2 | High |  |
| NGA | Nigeria | 10 | High |  |
| PAK | Pakistan | 6 | High |  |
| PHL | Philippines | 1 | High |  |
| POL | Poland | 2 |  |  |
| PRT | Portugal | 3 |  |  |
| RUS | Russia | 1 | High |  |
| LCA | Saint Lucia | 1 | Low |  |
| SEN | Senegal | 1 | High |  |
| SOM | Somalia | 7 | High |  |
| ZAF | South Africa | 1 | High |  |
| ESP | Spain | 1 |  | Travelled throughout Latin America |
| LKA | Sri Lanka | 1 |  |  |
| SWE | Sweden | 1 | Low | Exposure in high-risk community |
| TZA | Tanzania | 1 | High |  |
| THA | Thailand | 1 | High |  |
| TTO | Trinidad and Tobago | 1 |  | Travelled to St Vincent |
| TUR | Türkiye | 22 |  | Most from higher TB risk in SE Türkiye |
| UGA | Uganda | 3 | High |  |
| VNM | Viet Nam | 8 | High |  |
| ZWE | Zimbabwe | 1 | High |  |

*TB burden was defined as high if >150 incident cases and low if <10 incident cases per 100,000 inhabitants were reported any time between 2000-2018* [1,2]*.*

**Figure S1 – Tuberculosis diagnosis from the time since migration and recent travel in a migrant population in the London Borough of Hackney – among individuals who reported travel**

**
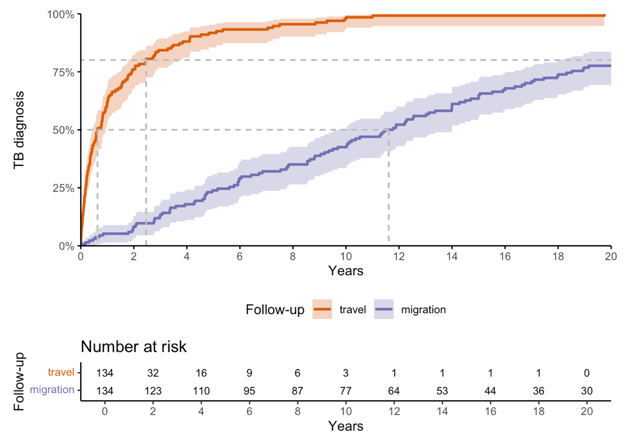
**

*Migration* *from and travel to a high-burden TB setting. TB, tuberculosis.*

**Figure S2 – Tuberculosis diagnosis from the time since migration and recent travel in a migrant population in the London Borough of Hackney – excluding individuals identified as part of active-case finding.**

**
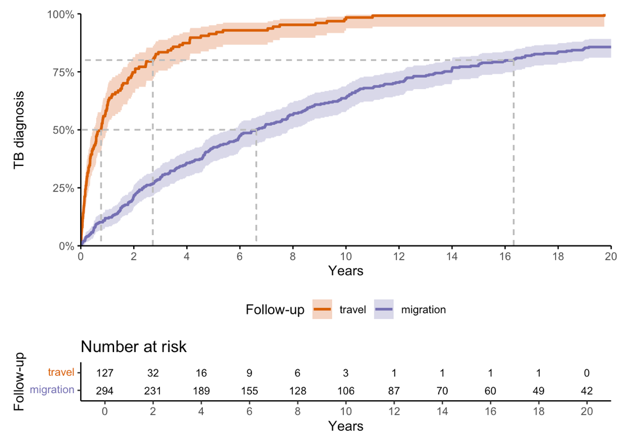
**

*Migration* *from and travel to a high-burden TB setting. Among those identified as part of active-case screening, 19 (73%) did not report recent travel. TB, tuberculosis.*

**Figure S3 – Tuberculosis diagnosis from the time since migration and recent travel in a migrant population in the London Borough of Hackney – including travel exclusively to a high TB burden country**

**
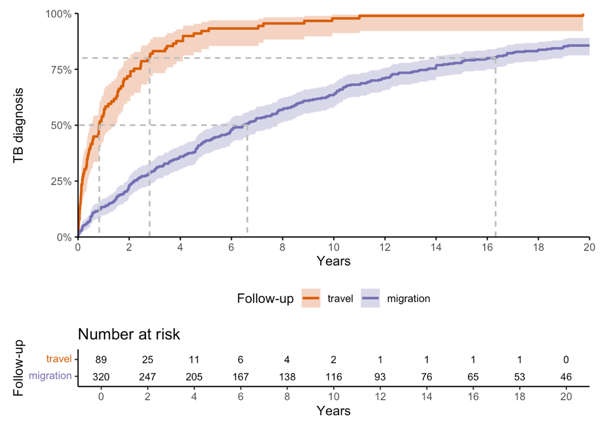
**

*Migration* *from and travel to a high TB burden country. TB, tuberculosis.*

**Figure S4 – Tuberculosis diagnosis from the time since migration and recent travel in a migrant population in the London Borough of Hackney – excluding travel to low TB burden countries**


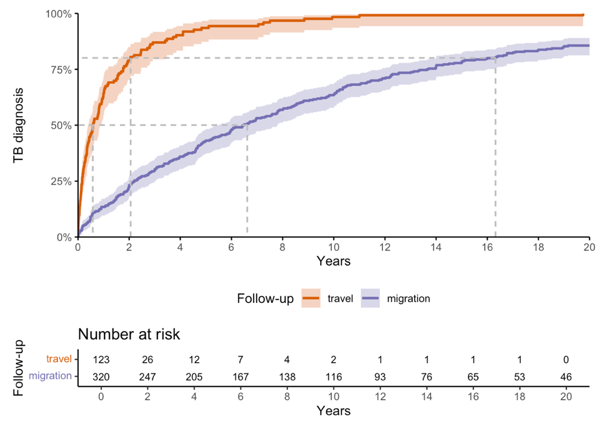


*Migration* *from and travel to a high-burden TB setting. TB, tuberculosis.*

**References:**

1. World Health Organization. Global Tuberculosis Report 2022. Geneva: WHO; 2022.

2. Lönnroth K, Migliori GB, Abubakar I, D’Ambrosio L, de Vries G, Diel R, et al. Towards tuberculosis elimination: an action framework for low-incidence countries. Eur Respir J. 2015;45: 928–952.
